# Association of Treated and Untreated Gastroesophageal Reflux Disease in the First Year of Life with the Subsequent Development of Asthma

**DOI:** 10.3390/ijerph18189633

**Published:** 2021-09-13

**Authors:** Anna Cantarutti, Claudio Barbiellini Amidei, Camilla Valsecchi, Antonio Scamarcia, Giovanni Corrao, Dario Gregori, Carlo Giaquinto, Jonas F. Ludvigsson, Cristina Canova

**Affiliations:** 1 National Centre for Healthcare Research and Pharmacoepidemiology, 20126 Milan, Italy; anna.cantarutti@unimib.it (A.C.); giovanni.corrao@unimib.it (G.C.); 2 Laboratory of Healthcare Research & Pharmacoepidemiology, Department of Statistics and Quantitative Methods, University of Milano-Bicocca, 20126 Milan, Italy; c.valsecchi22@campus.unimib.it; 3 Unit of Biostatistics, Epidemiology and Public Health, Department of Cardiac, Thoracic, Vascular Sciences and Public Health, University of Padova, 35131 Padova, Italy; claudioamidei@gmail.com (C.B.A.); dario.gregori@unipd.it (D.G.); 4 Pedianet Project, 25138 Padua, Italy; a.scamarcia@sosepe.com; 5 Division of Pediatric Infectious Diseases, Department of Woman’s and Child’s Health, University of Padua, 35131 Padua, Italy; carlo.giaquinto@unipd.it; 6 Department of Medical Epidemiology and Biostatistics, Karolinska Institutet, 171 77 Stockholm, Sweden; jonasludvigsson@yahoo.com; 7 Department of Pediatrics, Örebro University Hospital, 701 85 Örebro, Sweden; 8 Division of Epidemiology and Public Health, University of Nottingham School of Medicine, Nottingham NG7 2UH, UK; 9 Celiac Disease Center, Department of Medicine, Columbia University College of Physicians and Surgeons, New York, NY 10032, USA

**Keywords:** GERD, acid-suppressive medications, asthma, pediatric

## Abstract

Introduction: Gastroesophageal reflux disease (GERD) as well as its treatment with acid-suppressive medications have been considered possible risk factors for the development of asthma, but few studies have disentangled the role of GERD with that of its treatment. The present study aimed at estimating the association of treated and untreated GERD in the first year of life with the risk of asthma. Methods: Retrospective cohort study including all children born between 2004 and 2015 registered in Pedianet, an Italian primary care database. We analyzed the association of children exposed to GERD (both treated and untreated) in the first year of life with the risk of developing clinically assessed asthma (clinical asthma) after 3 years. Secondary outcomes included asthma identified by anti-asthmatic medications (treated asthma) and wheezing after 3 years. Hazard ratios (HR) and 95% confidence intervals (CI) were estimated comparing children with and without GERD, stratifying by treatment with acid-suppressive medications. Results: Out of 86,381 children, 1652 (1.9%) were affected by GERD in the first year of life, of which 871 (53%) were treated with acid-suppressive medications. Compared with controls, children with GERD were at increased risk of clinical asthma (HR: 1.40, 95% CI 1.15–1.70). Risks were similar between treated and untreated GERD (*p* = 0.41). Comparable results were found for treated asthma, but no risk increase was seen for wheezing. Discussion: Early-life GERD was associated with subsequent childhood asthma. Similar risks among children with treated and untreated GERD suggest that acid-suppressive medications are unlikely to play a major role in the development asthma.

## 1. Introduction

Gastric acid reflux is a common pediatric condition with a prevalence of about 50% in the first months of life that resolves spontaneously in 90% of cases before one year of age. In some cases, this condition may evolve into a more severe form of reflux defined as gastroesophageal reflux disease (GERD), which requires medical attention and can lead to severe consequences such as reflux esophagitis [1,2,3]. 

GERD has been considered a possible risk factor for the development of asthma, possibly due to a chronic irritation of the airway tract following the exposure to gastric acidity or due to effects of GERD treatment. Nevertheless, the precise pathophysiological mechanism by which any association with asthma might occur remains unclear.

GERD treatment may include the use of acid-suppressive medications, especially proton pump inhibitors (PPIs) and H2 receptor antagonists (H2RAs). Acid-suppressive medications, as well as antibiotics, are known to induce modifications of the human microbiome (dysbiosis) [4,5]. Evidence suggests that dysbiosis may have long-lasting effects that would be especially persistent among infants [6,7]. There is a growing interest in the possible role of dysbiosis on the development of several immune-mediated diseases, including asthma [8,9]. 

Three previous studies have reported an association between treatment with acid-suppressive medications (both PPIs and H2RAs) in early-life and asthma, with a 1.4- to 1.6-fold increased risk. This association was especially strong after treatment with PPIs, as well as suppressive medication in the first six months of life [10,11,12]. One study observed an association between PPIs and asthma among children, stratifying by GERD history. Nevertheless, exposure to PPIs was considered at any time in childhood and not in early life [11]. The main limitation of previous studies is confounding by indication, as none distinguished the effect of GERD from that of acid-suppressive medications in early life. Distinguishing GERD from acid-suppressive medication exposure is of great relevance given the possible causal role of GERD in the development of asthma [13,14]. 

Our study aimed at estimating the association of treated and untreated GERD in the first year of life on the subsequent development of asthma at 3 years, compared with children without GERD. 

## 2. Methods

### 2.1. Setting

**Figure 1:**
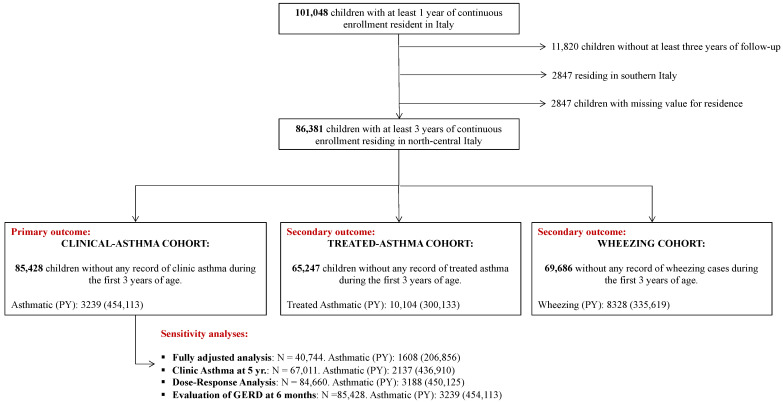
Flowchart of study cohorts. Pedianet. 2004–2015.

This retrospective cohort consisted of children born between 2004 and 2015, followed from birth to at least three years (maximum follow-up 14 years), assisted by a pediatrician within the Pedianet network resident in North or Central of Italy (Figure 1, Supplementary Materials—Table S1). 

Pedianet (http://www.pedianet.it/en accessed on 24 June 2021) is an independent network of family pediatricians (FPs) established in 1998 to collect information from outpatient routine clinical care in Italy. Such information includes reason for accessing healthcare, health status, demographic data, outpatient diagnoses and visits (free text or coded using the 9th International Statistical Classification of Diseases and Related Health Problems system (ICD-9 CM) codes), prescriptions (pharmaceutical prescriptions identified by the Anatomical Therapeutical Chemical (ATC) code), healthcare co-payment exemptions, specialist visits, diagnostic procedures, hospital admissions, growth parameters, and outcome data. Data generated during routine patient care were collected and handled anonymously, in compliance with Italian regulations, and stored under a unique numerical identifier.

Ethical approval of the study and the access to the database were approved by the Internal Scientific Committee of So.Se.Te. Srl, the legal owner of Pedianet.

### 2.2. Exposure to GERD

Exposure to GERD was defined by the presence in the first 12 months of life of a medical diagnosis of GERD or by the presence of ≥1 drug prescription of acid-suppressive medication that required a medical prescription with a diagnosis of GERD or suspected GERD and that needed to be dispensed by pharmacies (Supplementary Materials Figure S1). 

Pedianet was queried for any medical diagnosis of GERD (ICD-9-CM code 530.81) or free text in medical charts, by means of a string containing terms referring to GERD, including acronyms (in English and Italian) to maximize sensitivity. Any free text was then individually evaluated by a pediatrician. 

Children exposed to GERD were stratified based on drug prescription records. The treated GERD group referred to all children with at least one prescription of acid-suppressive medications in the first year of life, both PPIs (ATC code A02BC*) or H2RAs (ATC code A02BA*). The untreated GERD group comprised all children with a diagnosis of GERD but without any prescription of PPIs or H2RAs in the first year of life. Acid-suppressive medications require a pediatrician’s prescription to be dispensed by pharmacies for any pediatric treatment; therefore, we believe we identified all children treated for GERD. 

### 2.3. Outcomes

#### 2.3.1. Clinical Asthma

The primary outcome of interest was clinical asthma, defined as any reported medical diagnosis of asthma (ICD-9-CM code 493.*) or free text of medical charts based on a string containing terms (both in English and Italian such as “*asm*” or “*ast*”) referring to asthma. All results from the free-text search were then individually evaluated for a correct classification. Queries and possible diagnoses were not considered as asthma cases. All cases with a diagnosis of asthma before the age of 3 years were excluded from the main analyses that focused on incident asthma, a time when the diagnosis of asthma may be difficult to distinguish from transient wheezing that is not related to asthma (Figure 1).

We also considered as secondary outcomes of interest treated asthma and wheezing.

#### 2.3.2. Treated Asthma

Treated asthma was defined from Pedianet’s drug prescription records to identify all children with ≥2 prescriptions in a 12-month window that included short- and long-acting beta2 agonists (ATC code R03AC*), adrenergics in combination with other drugs—not anticholinergics—(ATC code R03AK*), inhaled corticosteroids (ATC code R03BA*), and antileukotriene drugs (ATC code R03DC*). This definition was based on a previously validated algorithm, with a positive predictive value of 78.5% and a sensitivity of 74.5%, that has also been used in a previous paper that investigated the association of GERD with asthma [10,15]. Treated asthma onset date was defined as the date of the first prescription in the 12-month frame. To increase the specificity of this algorithm, and for comparability with previous literature [10], any child with treated asthma onset before 3 years was excluded from these stratified analyses (Figure 1). 

#### 2.3.3. Wheezing

Wheezing was identified from medical charts using a string that included terms associated with wheezing (both in English and Italian such as “*ezing*”). This string was also tailored to maximize sensitivity, and all identified cases were then individually evaluated to include only confirmed medical diagnoses. All cases with a diagnosis of wheezing before the age of 3 were excluded from the asthma analyses (Figure 1).

### 2.4. Covariates 

Information on covariates used for confounding adjustment were obtained from outpatient diagnoses and visits and from prescription records. Since we focused on early-life exposures, our analyses included only covariates concerning the child’s exposure at birth and in the first year of life. We considered several baseline child characteristics that may affect the onset of asthma and wheezing: demographic variables (year of birth, gender, and region of birth), and healthcare utilization measures (including the number of distinct antibiotic treatments and the number of outpatient visits). For a subgroup of children, we further adjusted for maternal and delivery characteristics (gestational age, birth weight, and Apgar score at 1 min) since they are known risk factors for asthma [16]. 

### 2.5. Statistical Analyses

Descriptive statistics were provided for patients diagnosed with GERD (treated GERD and untreated GERD) and from children without GERD. Chi-squared test, Fisher’s test, and *Student’s t-test* were used for either categorical or continuous covariates to assess differences among children with or without GERD and among children with treated and untreated GERD.

### 2.6. Primary Outcome: Clinical Asthma

Follow-up of the cohort, including the incidence rate calculations, began at 3 years of age and ended with death, migration, change to a pediatrician outside of the Pedianet network, incident clinical asthma or end of follow-up (31 December 2017).

We used Cox regression models to estimate the hazard ratio (HR) and 95% confidence intervals (CIs) of the association between GERD (stratifying by treated GERD—any treatment and stratified by PPI and H2RA—and untreated GERD) with clinical asthma. Proportional hazards assumption was investigated by studying graphs over the log cumulative hazards function and Schoenfeld’s residuals. Nelson–Aalen cumulative hazards were used to plot the risk for the two exposures compared with children without GERD in the first year of life.

Results are reported according to two levels of adjustment. Model 1 was adjusted for year of birth, gender, and region of birth, and Model 2 also included number of visits to the pediatrician (≤5, 6–9, ≥10, as proxy of healthcare use) and antibiotic utilization in the first year of life (0, 1, 2, ≥3 antibiotic prescriptions).

### 2.7. Sensitivity and Subgroup Analyses 

Sensitivity and subgroup analyses were performed in order to evaluate the robustness of the main results. First, a dose–response analysis was performed; we considered the number of prescriptions of acid-suppressive medications (1, 2, or ≥3 prescriptions) and the subsequent development of asthma. The *p*-value for trend was evaluated running a model with the exposure variable as continuous. Second, we estimated risks with a fully adjusted model, also including maternal and delivery characteristics, only for 40,744 children without any missing data on such covariates. Third, to increase the specificity of the clinical asthma diagnosis, we evaluated incident clinical asthma at 5 years, given the higher possibility of having bronchospasms following respiratory infections among preschool children. All children with a diagnosis of asthma before the age of 5 were excluded from this analysis. 

Finally, we considered the risk of asthma following exposure to GERD in the first 6 months of life. 

### 2.8. Secondary Outcomes

Cox regression models were fitted for estimating hazard ratios (HRs), and corresponding 95% confidence intervals (CIs) for the association between GERD and treated asthma and wheezing, respectively. Reported results were adjusted according to Model 2.

## 3. Results

Out of 86,381 children residing in the northern or central part of Italy, with ≥3 years of follow up, 1652 children (1.9%) had a diagnosis of GERD in the first year of life: 871 (53%) had ≥1 acid-suppressive medication (treated GERD), while 781 had ≥1 medical diagnosis of GERD without any prescription of PPIs or H2RAs (untreated GERD). Baseline characteristics of the children in the cohort, as well as their healthcare utilization and maternal and delivery characteristics stratified by GERD exposure are reported in Table 1.

There were substantial differences in the baseline characteristics of children with GERD compared with those without GERD and between treated and untreated GERD. Children with GERD, and especially treated GERD children, were more likely to redeem prescriptions for antibiotics, to have more medical visits, and to have a shorter gestation and lower birth weight. 

### 3.1. Clinical Asthma 

**Figure 2:**
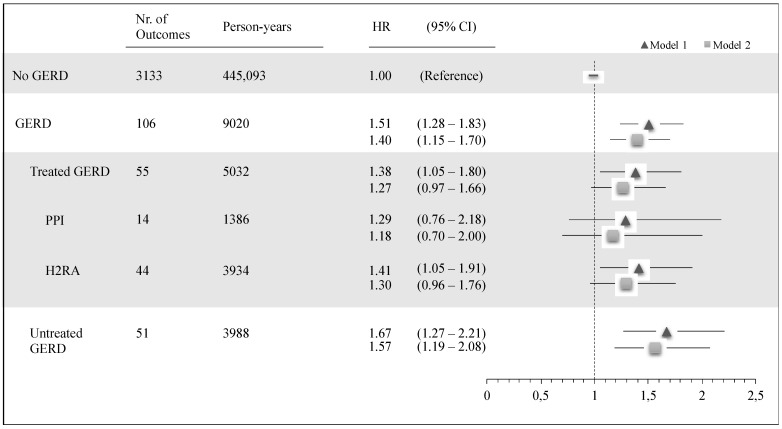
Association of clinical asthma with GERD (treated and untreated) compared with children without GERD. Pedianet, 2004–2015. N = 85,428; Model 1: adjusted for sex, region of birth, and year of birth; Model 2: adjusted for sex, region of birth, year of birth, number of medical visits to the pediatrician, and number of antibiotics utilized in the first year of life.

85,428 children were included in the clinical asthma cohort to assess asthma onset at 3 years or later (total follow-up beyond age 3 years was 454,113 person-years). All characteristics apart from sex were unevenly distributed among children with and without GERD and among treated and untreated GERD (Table S2). Among the 83,804 children without GERD, 3133 (3.7%) developed asthma, compared with 106 of 1624 children with GERD (6.5%). Model 1 and Model 2 adjusted HRs for clinical asthma stratified for all exposures of interest are reported in Figure 2. Children with GERD showed an increased risk of clinical asthma both in the first level of adjustment (Model 1 HR: 1.51, 95% CI 1.24–1.83) and in the second level of adjustment (Model 2: 1.40, 1.15–1.70) compared with those without GERD. Similar results were seen in treated and untreated GERD, although not all risk estimates attained statistical significance (Supplementary Materials Figure S2). 

**Figure 3:**
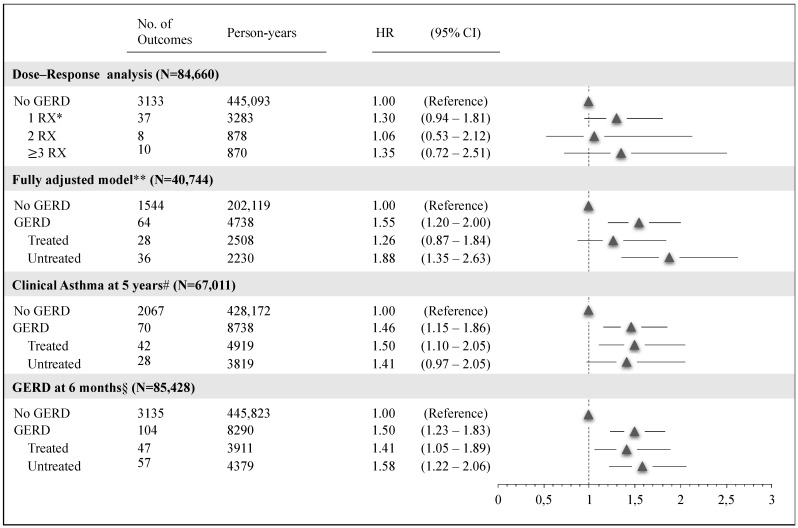
Sensitivity analyses. Adjusted hazard ratio and 95% CI for clinical asthma. Pedianet, 2004–2015. * RX: number of prescriptions of acid-suppressive medications. ** Fully adjusted model included: sex, region of birth, year of birth, number of medical visits to the pediatrician, number of antibiotics utilized in the first year of life, gestational age, birth weight, and Apgar score at 1 min; # Clinical diagnosis of asthma at 5 years (incident cases). § Exposure to GERD in the first 6 months of life.

The results of sensitivity and subgroup analyses are summarized in Figure 3. GERD was associated with increased risks of clinical asthma in all the sensitivity and subgroup analyses (Tables S7–S9). We found no association between the number of redeemed acid-suppressive medications and clinical asthma (*p* = 0.14) (Table S6). Moreover, when clinical asthma was evaluated at 5 years or later, we observed a similarly significant increased risk of clinical asthma in children with treated GERD (HR: 1.50, 95% CI 1.10–2.05) compared with children without GERD (Table S8). When we evaluated GERD in the first 6 months of life, both treated GERD (HR: 1.41, 95% CI 1.05–1.89) and untreated GERD (HR: 1.58, 95% CI 1.22–2.06) were associated with an increased risk of clinical asthma (Table S9). 

### 3.2. Treated Asthma and Wheezing

Out of 86,381 children, 65,247 were included in the treated asthma cohort to assess treated asthma onset after 3 years of age (with a 300,133 person-year follow-up). All birth characteristics are reported in Tables S3 and S4. Among children without GERD, 9862 (15%) had treated asthma, compared with 242 (22.5%) children with GERD.

When the outcome of interest was wheezing, we restricted the cohort to 69,686 children without any records of wheezing in the first three years of life (335,619 person-years). Among children without GERD, 8151 (12%) had a diagnosis of wheezing, compared with 177 (10%) children with GERD.

**Figure 4:**
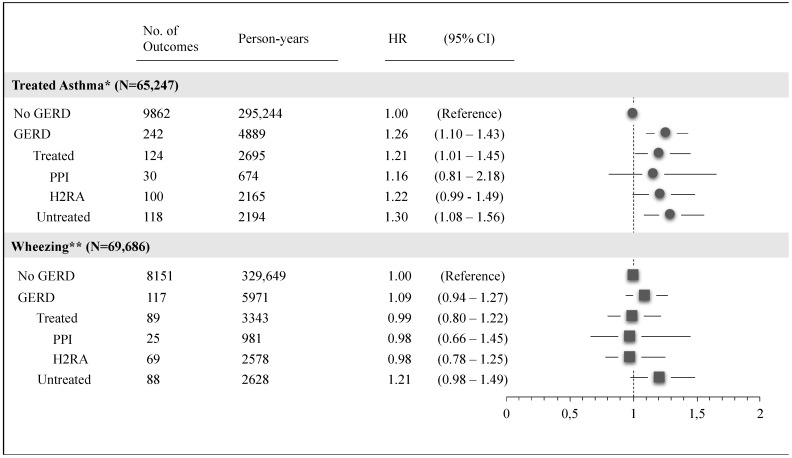
Adjusted hazard ratio and 95% CI for treated asthma and wheezing, according to the different level of GERD exposure. Pedianet, 2004–2015. * Treated asthma: incident cases identified by anti-asthmatic medications. ** Wheezing: incident cases identified by clinical diagnosis of wheezing.

Figure 4 shows the association with secondary outcomes of treated asthma and wheezing. Children with GERD had a significantly increased risk of treated asthma compared with children without GERD (HRs were 1.26, 1.21, and 1.30, respectively, for GERD, treated GERD, and untreated GERD). In contrast, we found no association between GERD and wheezing. We found consistency with these results across all sensitivity analyses (Tables S10 and S11). 

## 4. Discussion

Our results suggest that exposure to GERD in the first year of life is associated with a marked increase in asthma onset during childhood, but no significant difference between treated and untreated GERD was observed. 

Sensitivity and subgroup analyses conducted to address residual confounding (i.e., fully adjusted model) and to increase the specificity of asthma diagnosis (onset after 5 years) and of exposure to GERD in the first 6 months of life were coherent with the main findings of this study. 

To the best of our knowledge, only few studies in the literature have previously investigated the association between acid-suppressive medication exposure in the first year of life and asthma onset [10,11,12]. Acid-suppressive medications are almost exclusively administered to infants for GERD treatment. GERD has also been considered a possible risk factor for the development of asthma, probably due to chronic irritation of the airway tract that is exposed to gastric acidity. Nevertheless, the fact that GERD is associated with the subsequent development of asthma has not been fully established, and evidence from previous studies has shown contrasting results. Our study suggests an increased risk of asthma among children is associated with GERD. This is not consistent with the findings of a previous study that concluded GERD in infants seems to be associated only with an acute irritation of the bronchi that would cause bronchospasms but would not lead to the development of asthma later on in life [13]. GERD could also be associated with other atopic conditions, so children with GERD might be more prone to develop asthma [17,18]. 

Some studies have suggested that a longer history of GERD would be associated with the development of more severe asthma [19], but results have been inconsistent [20,21]. Our results do not support this hypothesis, as we found similar risks following exposure to GERD in the first 6 and 12 months of life.

A systematic review has reported an elevated prevalence of GERD among children with asthma (OR: 5.6 95% CI 4.3–6.9), but it is not clear if this association is causal [14].

Several hypotheses in the literature have separately linked GERD and acid-suppressive medications with the development of asthma. Most hypotheses that provided a causal explanation for exposure to acid-suppressive medications are related to dysbiosis. Dysbiosis would lead to an alteration of the immune system following a modified protein’s digestion, due to higher gastric pH, thereby promoting altered immune system trajectories, with a greater risk of developing allergic disorders [22]. Given their possible side-effects, evidence in the literature recommends avoiding acid-suppressive medications to treat GERD in infants [23,24,25,26]. Considering the relevance of asthma and the potential harm of acid-suppressive medications, it would always be advisable to evaluate the actual benefits derived from this type of treatment before prescribing these medications to infants with GERD.

GERD itself is responsible for bronchospasms and inflammation of the airway tract; due to frequent acid aspiration, this condition induces nerve stimulation with bronchospasms that could possibly lead to the development of asthma [27]. The consistency of our findings, after excluding incident asthma before the age of three and therefore better separating exposure and outcome, are suggestive of a possible causal relationship with long-term consequences to the airway tract following exposure to GERD. 

### Strengths and Limitations

This was one of the first large-scale studies that separately analyzed the association of GERD treatment, and that of clinically assessed GERD without treatment in early life, with the risk of developing asthma. The lack of a clinical assessment of the exposure was the main limitation of most previous studies that only focused on exposure to acid-suppressive medications [10,12]. Analyses on wheezing as an outcome have also not been taken into account by previous studies. Another point of strength is the robustness of our results across all our sensitivity and subgroup analyses. We also observed consistent results between our two different definitions of outcome (clinical asthma and treated asthma), that confirm the solidity of our findings with a very specific and a very sensitive definition of asthma. The prevalence of asthma was furthermore in line with that reported in the previous literature [10,28], as was the magnitude of the risk of asthma among infants treated with acid-suppressive medications, ranging from 1.62 (1.47–1.78) [10] to risks reported by Mitre et al. for H2RAs of 1.25 (1.21–1.29) and PPIs of 1.41 (1.31–1.52) [12] and those reported by Wang et al. for exposure to PPIs in the first 6 months of life of 1.83 (1.65–2.03) [11]. In contrast with previous studies, we did not observe significantly higher risks associated with PPI exposure. This could be due to the low number of children exposed to PPIs in our study, or residual confounding that we did not take into account. 

Nevertheless, we found no significantly increased risk of asthma among children with treated GERD compared with untreated GERD. Similarly, Wang et al. did not find any significant difference according to earlier history of GERD when they examined PPIs and risk of asthma (*p* for interaction 0.82) [11]. Anyway, there are limits to the comparability of these results due to different research objectives, exposure in early life vs. childhood-long exposure in Wang’s study (cohort mean age at exposure to PPIs was 12.9 years), and the absence of a specific time frame when GERD occurred [11]. Furthermore, when analyzing risks associated with PPIs among children with a previous history of GERD, Wang’s study did not assess the possible bias of active GERD, treated with PPIs, that could be responsible for acute asthma exacerbations, while in our study only eight children (5.1%) exposed to GERD in their first year of life were affected by GERD the year before asthma onset [11]. 

Another strength of our study is its separation of GERD exposure from all considered outcomes (≥2 years apart), thereby reducing this possible risk of misclassification. The analysis of clinically diagnosed asthma among children 5 years or older reduced the risk of including children with bronchospasms often caused by viral infections that are common below 5 years of age, or including other non-asthmatic conditions [16,28].

The limitations of our study relate to the limited statistical power in some stratified analyses, such as that on dose–response that were not suggestive of any trend for the number of prescriptions of acid-suppressive medications, unlike what has been observed by previous studies (Supplementary Materials Table S12) [10,11]. The absence of analyses on duration of GERD and clinical assessment of GERD severity did not allow us to further stratify our analyses, and this should be addressed by future studies. Infants that were prescribed acid-suppressive medications could in fact be affected by more severe GERD, compared with those with a diagnosis alone. However, we did not find an increased risk of asthma when comparing children with treated GERD with those with untreated GERD, suggesting these medications are unlikely to play a role in the development of asthma (Supplementary Materials Table S13). Despite adjusting for the number of antibiotic prescriptions that are known to induce modifications of the human microbiome and that may promote the development of several immune-mediated diseases, including asthma, and that resulted in being associated with both treated and untreated GERD (Table 1), we were not able to take into account infective diseases as well as other atopic conditions with onset prior or subsequent to that of GERD and that may contribute to explaining the association we observed between GERD and asthma. Furthermore, milk protein allergy is characterized by symptoms that are difficult to distinguish from those of GERD, and the absence of data on the use of protein hydrolysate formula or the elimination of cow’s milk from maternal diet for breastfeeding or clinical signs associated with milk protein allergy did not allow us to investigate the role of this possible confounder. Incomplete data on maternal and delivery covariates allowed us to perform fully adjusted analyses only on a subgroup of children, but we observed similar results to those with basic adjustment. Due to missing data on parental smoking habits, asthma and immune-mediated conditions in parents and siblings, maternal drug prescriptions during pregnancy (especially to acid-suppressive medications), environmental indoor and outdoor exposures, respiratory infections, genotypic characteristics and type of delivery, and healthcare utilization (including hospitalizations and emergency department visits), we were not able to take into account these potential confounders. 

## 5. Conclusions

We observed an increased risk of clinical asthma among infants with GERD in early-life, compared with children without GERD. Similar risk estimates in children with treated and untreated GERD indicate that acid-suppressive medications are unlikely to play a major role in childhood asthma development. 

## Figures and Tables

**Table 1:** Demographic and clinical characteristics. Study cohort. Pedianet, 2004–2015, N = 86,381.

	GERD	GERD		No GERD	*p*-Value	
		Treated	Untreated		GERD vs. No GERD	Treated vs. Untreated
	(N = 1652)	(N = 871)	(N = 781)	(N = 84,729)		
Year of birth						
2004–2007	509 (30.8)	267 (30.7)	242 (31.0)	29,767 (35.1)	0.0011	<0.0001
2008–2011	575 (34.8)	367 (42.1)	208 (26.6)	28,159 (33.2)		
2012–2015	568 (34.4)	237 (27.2)	331 (42.4)	26,803 (31.6)		
Gender						
Female	785 (47.5)	418 (48.0)	367 (47.0)	40,974 (48.4)	0.4983	0.6846
Male	867 (52.5)	453 (52.0)	414 (53.0)	43,755 (51.6)		
No. of Antibiotics						
0	803 (48.6)	404 (46.4)	339 (51.1)	54,923 (64.8)	<0.0001	0.0047
1	402 (24.3)	201 (23.1)	201 (25.7)	15,491 (18.3)		
2	208 (12.6)	117 (13.4)	91 (11.6)	7442 (8.8)		
≥3	239 (14.5)	149 (17.1)	90 (11.5)	6873 (8.1)		
No. of medical visits—mean (STD)	14.92 (6.2)	15.57 (6.6)	14.20 (5.7)	9.09 (5.8)	<0.0001	<0.0001
≤ 5	38 (2.3)	16 (1.8)	22 (2.8)	20,157 (23.8)	<0.0001	0.0273
6–9	247 (14.9)	114 (13.1)	133 (17.0)	26,669 (31.5)		
≥10	1367 (82.8)	741 (85.1)	626 (80.2)	37,903 (44.7)		
Gestational age—mean (STD) ⱡ	38.70 (1.9)	38.50 (2.0)	38.90 (1.8)	39.03 (1.7)	<0.0001	0.0012
≤28 GW	0 (0.0)	0 (0.0)	0 (0.0)	41 (0.1)	<0.0001	0.3814
29–35 GW	54 (5.9)	30 (6.6)	24 (5.3)	1341 (3.3)		
≥36 GW	854 (94.1)	422 (93.4)	432 (94.7)	39,015 (96.6)		
Birth weight—mean (STD) ⱡ	3158.15 (523.7)	3120.76 (546.1)	3195.21 (498.3)	3276.47 (498.6)	<0.0001	0.0321
<2500 gr.	82 (9.0)	51 (11.3)	31 (6.8)	2345 (5.8)	<0.0001	0.0184
≥2500 gr.	826 (91.0)	401 (88.7)	425 (93.2)	38,052 (94.2)		
Apgar 1 score—mean (STD) ⱡ	8.86 (1.0)	8.81 (1.1)	8.91 (0.8)	8.95 (0.9)	0.0038	0.1577
<7	28 (3.1)	19 (4.2)	9 (2.0)	1021 (2.5)	0.2920	0.0520
≥7	880 (96.9)	433 (95.8)	477 (98.0)	39,376 (97.5)		

ⱡ Missing data are handled in the analysis N = 45,076.

## Data Availability

Anna Cantarutti and Cristina Canova had full access to all the data in the study and take responsibility for the integrity of the data and the accuracy of the data analysis. Anna Cantarutti (Laboratory of Healthcare Research & Pharmacoepidemiology, Department of Statistics and Quantitative Methods, University of Milano-Bicocca, Milan, Italy), Camilla Valsecchi (Laboratory of Healthcare Research & Pharmacoepidemiology, Department of Statistics and Quantitative Methods, University of Milano-Bicocca, Milan, Italy), and Cristina Canova (Unit of Biostatistics, Epidemiology and Public Health, Department of Cardiac, Thoracic, Vascular Sciences and Public Health, University of Padova, Padova, Italy) conducted and are responsible for the data analysis.

## Supplementary Materials

The following are available online at https://www.mdpi.com/1660-4601/18/18/9633/s1, Table S1. North-Central Italian regions included in the study. Pedianet, 2004–2015. Table S2. Demographic and clinical characteristics. Clinical-Asthma cohort. Pedianet, 2004–2015. N = 85,428. Table S3. Demographic and clinical characteristics. Treated-Asthma cohort. Pedianet, 2004–2015. N = 65,247. Table S4. Demographic and clinical characteristics. Wheezing cohort. Pedianet, 2004–2015. N = 69,695. Table S5. Adjusted hazard ratio and 95% CI for clinical-asthma, according to the different level of GERD exposure. Pedianet, 2004–2015. N = 85,428. Table S6. Dose-response Sensitivity analysis. Adjusted hazard ratio and 95% CI for clinical-asthma, according to the different level of GERD exposure. Pedianet, 2004–2015. N = 85,428. Table S7. Sensitivity analysis. Full-Adjusted hazard ratio and 95% CI for clinical-asthma stratified by the presence of GERD history and further stratified by treatment with acid-suppressive medications according to the different level of GERD exposure. Pedianet, 2004–2015. N = 40,744. Table S8. Clinical-Asthma at 5 years Sensitivity analysis. Adjusted hazard ratio and 95% CI for clinical-asthma, according to the different level of GERD exposure. Pedianet, 2004–2015. N = 67,011. Table S9. Sensitivity analysis. Adjusted hazard ratio and 95% CI for clinical-asthma, according to the different level of GERD exposure evaluated in the first 6 month of life. Pedianet, 2004–2015. N = 85,428. Table S10. Adjusted hazard ratio and 95% CI for treated-asthma, according to the different level of GERD exposure. Pedianet, 2004–2015. N = 65,247. Table S11. Adjusted hazard ratio and 95% CI for wheezing, according to the different level of GERD exposure. Pedianet, 2004–2015. N = 69,686. Table S12. Distribution of monotherapy and politherapy acid-suppressive medications. Pedianet, 2004–2015. Table S13. Severity of GERD. Adjusted hazard ratio and 95% CI. Pedianet, 2004–2015. Figure S1. Definition of exposure and outcomes. Pedianet, 2004–2015. Figure S2. Nelson-Aalen cumulative hazards for the development of childhood asthma following exposure to GERD in the first year of life, compared to no GERD children.
