# Supplementary material for: Association of Treated and Untreated Gastroesophageal Reflux Disease in the First Year of Life with the Subsequent Development of Asthma
Source: Int J Environ Res Public Health. 2021 Sep 13;18(18):9633. doi: 10.3390/ijerph18189633 (PMC8468622; doi:10.3390/ijerph18189633)
Supplement: Supplementary file 1 [file ijerph-18-09633-s001.zip › ijerph-1292033-supplementary.pdf]

Supplementary Materials for

Association of treated and untreated gastroesophageal reflux disease in the first year of life with the subsequent development of asthma.

Anna Cantarutti, Claudio Barbiellini Amidei , Camilla Valsecchi, Antonio Scamarcia, Giovanni Corrao, Dario Gregori, Carlo Giaquinto, Jonas F Ludvigsson, Cristina Canova

Correspondence to: Cristina Canova, Unit of Biostatistics, Epidemiology and Public Health, Department of Cardiac, Thoracic, Vascular Sciences and Public Health, University of Padova, Padova, Italy. Email: cristina.canova@unipd.it

Table S1. North-Central Italian regions included in the study. Pedianet, 2004-2015.

Piedmont
Lombardy
Trentino- Alto Adige/Südtirol
Friuli-Venezia Giulia
Veneto
Emilia-Romagna
Liguria
Tuscany
Marche
Umbria
Lazio

Table S2. Demographic and clinical characteristics. Clinical-Asthma cohort. Pedianet, 2004-2015. N=85,428

		GERD (N=1624)	GERD Treated (N=856)	GERD Untreated (N=768)	No GERD (N=83,804)	p-value GERD vs. No GERD	p-value Treated vs. Untreated
Year of birth							
	2004 - 2007	500 (30·8)	260 (30·4)	240 (31·3)	29395 (35·1)	0·0013	<·0001
	2008 - 2011	565 (34·8)	361 (42·2)	204 (26·6)	27885 (33·3)		
	2012 - 2015	559 (34·4)	235 (27·5)	324 (42·2)	26524 (31·7)		
Gender							
	Female	778 (47·9)	415 (48·5)	363 (47·3)	40658 (48·5)	0·6266	0·6244
	Male	846 (52·1)	441 (51·5)	405 (52·7)	43146 (51·5)		
Nr· of Antibiotics							
	0	793 (48·8)	400 (46·7)	393 (51·2)	54555 (65·1)	<·0001	0·0058
	1	396 (24·4)	198 (23·1)	198 (25·8)	15256 (18·2)		
	2	205 (12·6)	114 (13·3)	91 (11·8)	7313 (8·7)		
	≥ 3	230 (14·2)	144 (16·8)	86 (11·2)	6680 (8·0)		
Nr· of medical visits - mean (STD)		14·87 (6·2)	15·49 (6·6)	14·18 (5·7)	9·04 (5·7)	<·0001	0·0109
	0 - 5	38 (2·3)	16 (1·9)	22 (2·9)	20093 (24·0)	<·0001	0·0375
	6 - 9	245 (15·1)	114 (13·3)	131 (17·1)	26475 (31·6)		
	≥ 10	1341 (82·6)	726 (84·8)	615 (80·1)	37236 (44·4)		
Gestational age - mean (STD)ɫ		38·69 (1·9)	38·49 (2·0)	38·88 (1·8)	39·03 (1·7)	<·0001	0·0019
	<= 28 GW	0 (0·0)	0 (0·0)	0 (0·0)	40 (0·1)	<·0001	0·4683
	29 - 35 GW	53 (6·0)	29 (6·6)	24 (5·4)	1312 (3·3)		
	>= 36 GW	835 (94·0)	414 (93·5)	421 (94·6)	38504 (96·6)		
Birth weight - mean (STD)ɫ		3157·84 (524·3)	3122·33 (549·0)	3193·18 (496·6)	3276·99 (498·4)	<·0001	0·0440
	< 2500gr·	80 (9·0)	50 (11·3)	30 (6·7)	2298 (5·8)	<·0001	0·0180
	≥ 2500gr·	808 (91·0)	393 (88·7)	415 (93·3)	37558 (94·2)		
Apgar 1 score - mean (STD)ɫ		8·86 (1·0)	8·81 (1·2)	8·91 (0·8)	8·95 (0·9)	0·0033	0·1591
	< 7	28 (3·2)	19 (4·3)	9 (2·0)	1000 (2·5)	0·2261	0·0533
	≥ 7	860 (96·9)	424 (95·7)	436 (98·0)	38856 (97·5)		

ɫ Missing data are handled in the analysis N=44,684

Table S3. Demographic and clinical characteristics. Treated-Asthma cohort. Pedianet, 2004-2015. N=65,247

	GERD	GERD		No GERD	p-value	
	(N=1075)	Treated (N=543)	Untreated (N=532)	(N=64,172)	GERD vs. No GERD	Treated vs. Untreated
Year of birth					<.0001	<.0001
2004 - 2007	326 (30·3)	168 (30·9)	158 (29·7)	23499 (36·6)		
2008 - 2011	367 (34·1)	221 (40·7)	146 (27·4)	20790 (32·4)		
2012 - 2015	382 (35·5)	154 (28·4)	228 (42·9)	19883 (31·0)		
Gender					0·9218	0·3143
Female	538 (50·0)	280 (51·6)	258 (48·5)	32019 (49·9)		
Male	537 (49·9)	263 (48·4)	274 (51·5)	32153 (50·1)		
Nr· of Antibiotics					<·0001	0·3972
0	596 (55·4)	293 (54·0)	303 (57·0)	46509 (72·5)		
1	267 (24·8)	136 (25·1)	131 (24·6)	10558 (16·5)		
2	109 (10·1)	54 (9·9)	55 (10·3)	4210 (6·6)		
≥ 3	103 (9·6)	60 (11·1)	43 (8·1)	2895 (4·5)		
Nr· of medical visits - mean (STD)	13·78 (5·6)	14·17 (5·9)	13·39 (5·2)	7·98 (5·3)	<·0001	<·0001
0 -- 5	34 (3·2)	14 (2·6)	20 (3·8)	18623 (29·0)	<·0001	0·2132
6 -- 9	194 (18·1)	90 (16·5)	104 (19·6)	21897 (34·1)		
≥ 10	847 (78·8)	493 (80·9)	408 (76·7)	23652 (36·9)		
Gestational age - mean (STD)ł	38·72 (1·9)	38·55 (2·0)	38·88 (1·8)	39·07 (1·64)	<·0001	0·0309
<= 28 GW	0 (0·0)	0 (0·0)	0 (0·0)	22 (0·1)	0·0022	0·6129
29 - 35 GW	32 (5·6)	17 (6·1)	15 (5·1)	906 (3·1)		
>= 36 GW	542 (94·4)	263 (93·9)	279 (94·9)	28657 (96·9)		
Birth weight - mean (STD)ł	3150·73 (521·6)	3109·79 (537·6)	3189·72 (503·8)	3276·59 (493·1)	<·0001	0·0664
< 2500gr·	50 (8·7)	32 (11·4)	18 (6·1)	1685 (5·7)	0·0021	0·0242
≥ 2500gr·	524 (91·3)	248 (88·6)	276 (93·9)	27900 (94·3)		
Apgar 1 score - mean (STD)ł	8·84 (1·0)	8·78 (1·2)	8·90 (0·8)	8·97 (0·9)	0·0016	0·1451
< 7	21 (3·7)	14 (5·0)	7 (2·4)	724 (2·5)	0·0640	0·0948
≥ 7	553 (96·3)	266 (95·00)	287 (97·6)	28861 (97·6)		

ł Missing data are handled in the analysis N=35,088

Table S4. Demographic and clinical characteristics. Wheezing cohort. Pedianet, 2004-2015. N=69,695

	GERD	GERD		No GERD	p-value	
		Treated	Untreated		GERD vs· No GERD	Treated vs· Untreated
	(N=1197)	(N=633)	(N=564)	(N=68'489)		
Year of birth						
2004 - 2007	366 (30·6)	191 (30·2)	175 (31·0)	24379 (35·6)	0·0010	<·0001
2008 - 2011	412 (34·4)	259 (40·9)	153 (27·1)	22532 (32·9)		
2012 - 2015	419 (35·0)	183 (28·9)	236 (41·8)	21578 (31·5)		
Gender						
Female	614 (51·3)	322 (50·9)	292 (51·8)	34413 (50·3)	0·4718	0·7547
Male	583 (48·7)	311 (49·1)	272 (48·2)	34076 (49·8)		
Nr· of Antibiotics						
0	647 (54·1)	325 (51·3)	322 (57·1)	47915 (67·0)	<·0001	0·0209
1	285 (23·8)	149 (23·5)	136 (24·1)	11477 (16·8)		
2	129 (10·8)	71 (11·2)	58 (10·3)	5036 (7·4)		
≥ 3	136 (11·4)	88 (13·9)	48 (8·5)	4061 (5·9)		
Nr· of medical visits - mean (STD)	14·15 (5·9)	14·81 (6·0)	13·40 (5·2)	8·34 (5·5)	<·0001	<·0001
0 -- 5	33 (2·8)	15 (2·4)	18 (3·2)	18914 (27·6)	<·0001	0·0593
6 -- 9	210 (17·5)	97 (15·3)	113 (20·0)	22508 (32·9)		
≥ 10	954 (79·7)	521 (82·3)	433 (76·8)	27067 (39·5)		
Gestational age - mean (STD)ɫ	38·78 (1·8)	38·56 (1·9)	38·98 (1·8)	39·07 (1·6)	<·0001	0·0035
<= 28 GW	0 (0·0)	0 (0·0)	0 (0·0)	25 (0·1)	0·0109	0·4243
29 - 35 GW	32 (5·1)	18 (5·8)	14 (4·4)	939 (3·0)		
>= 36 GW	602 (95·0)	295 (94·3)	307 (95·6)	30072 (96·9)		
Birth weight - mean (STD)ɫ	3167·97 (510·2)	3118·65 (516·9)	3216·05 (499·8)	3276·75 (491·4)	<·0001	0·0161
< 2500gr·	52 (8·2)	34 (10·9)	18 (5·6)	1730 (5·6)	0·0045	0·0159
≥ 2500gr·	582 (91·8)	279 (89·1)	303 (94·4)	29306 (94·4)		
Apgar 1 score - mean (STD)ɫ	8·86 (1·0)	8·77 (1·3)	8·95 (0·7)	9·96 (0·9)	0·0095	0·0210
< 7	19 (3·0)	17 (5·4)	2 (0·6)	767 (2·5)	0·3998	0·0004
≥ 7	615 (97·0)	296 (94·6)	319 (99·4)	30269 (97·5)		

ɫ Missing data are handled in the analysis N=38,016

Table S5. Adjusted hazard ratio and 95% CI for clinical-asthma, according to the different level of GERD exposure. Pedianet, 2004-2015. N=85,428

	Model 1		Model 2	
	HR	(95% CI)	HR	(95% CI)
No Gerd	(Reference)		(Reference)	
GERD	1·51	(1·24 - 1·83)	1·40	(1·15 - 1·70)
Treated	1·38	(1·05 - 1·8)	1·27	(0·97 - 1·66)
PPI	1·29	(0·76 - 2·18)	1·18	(0·70 - 2·00)
H2RA	1·41	(1·05 - 1·91)	1·30	(0·96 - 1·76)
Untreated	1·67	(1·27 - 2·21)	1·57	(1·19 - 2·08)

Table S6. Dose-response Sensitivity analysis. Adjusted hazard ratio and 95% CI for clinical-asthma, according to the different level of GERD exposure. Pedianet, 2004-2015. N=85,428

	HRƗ	95% CI	p-trend
No Gerd	(Reference)		0.14
GERD			
Treated	1·27	(0·97 - 1·66)	
1	1·30	(0·94 - 1·81)	
2	1·06	(0·53 - 2·12)	
≥3	1·35	(0·72 - 2·51)	

Ɨ Adjusted for: Gender, Year of birth, Region, Number of antibiotic prescriptions in the first year of life, Number of outpatient visits

Table S7. Sensitivity analysis. Full-Adjusted hazard ratio and 95% CI for clinical-asthma stratified by the presence of GERD history and further stratified by treatment with acid-suppressive medications according to the different level of GERD exposure. Pedianet, 2004-2015. N= 40,744

	HR	(95% CI)
No Gerd	(Reference)	
GERD	1·55	(1·20 - 2·00)
Treated	1·26	(0·87 - 1·84)
Untreated	1·88	(1·35 - 2·63)

Table S8. Clinical-Asthma at 5 years Sensitivity analysis. Adjusted hazard ratio and 95% CI for clinical-asthma, according to the different level of GERD exposure. Pedianet, 2004-2015. N=67,011

	HRƗ	95% CI
No Gerd	(Reference)	
GERD	1·46	(1·15 - 1·86)
Treated	1·50	(1·10 - 2·05)
Untreat ed	1·41	(0·97 - 2·05)

Ɨ Adjusted for: Gender, Year of birth, Region, Number of antibiotic prescriptions in the first year of life, Number of outpatient visits

Table S9. Sensitivity analysis. Adjusted hazard ratio and 95% CI for clinical-asthma, according to the different level of GERD exposure evaluated in the first 6 month of life. Pedianet, 2004-2015. N=85,428

	HRƗ	95% CI
No Gerd	(Reference)	
GERD	1.50	(1.23 - 1.83)
Treated	1.41	(1.05 - 1.89)
Untreated	1.58	(1.22 - 2.06)

Ɨ Adjusted for: Gender, Year of birth, Region, Number of antibiotic prescriptions in the first year of life, Number of outpatient visits

Table S10. Adjusted hazard ratio and 95% CI for treated-asthma, according to the different level of GERD exposure. Pedianet, 2004-2015. N=65,247

	HRƗ	95% CI
No Gerd	(Reference)	
GERD	1·26	(1·10 - 1·43)
Treated	1·21	(1·01 - 1·45)
PPI	1·16	(0·81 - 1·66)
H2RA	1·22	(0·99 - 1·49)
Untreated	1·30	(1·08 - 1·56)

Ɨ Adjusted for: Gender, Year of birth, Region, Number of antibiotic prescriptions in the first year of life, Number of outpatient visits

Table S11. Adjusted hazard ratio and 95% CI for wheezing, according to the different level of GERD exposure. Pedianet, 2004-2015. N=69,686

	HRƗ	95% CI
No Gerd	(Reference)	
GERD	1·09	(0·94 - 1·27)
Treated	0·99	(0·80 - 1·22)
PPI	0·98	(0·66 - 1·45)
H2RA	0·98	(0·78 - 1·25)
Untreated	1·21	(0·98 - 1·49)

Ɨ Adjusted for: Gender, Year of birth, Region, Number of antibiotic prescriptions in the first year of life, Number of outpatient visits

Table S12. Distribution of monotherapy and politherapy acid-suppressive medications. Pedianet, 2004-2015.

Clinical- **Asthma**		N
Treated GERD		856
	PPI	185
	H2RA	624

	PPI & H2RA	47

**Treated-Asthma**		
Treated GERD		543
	PPI	111
	H2RA	404
	PPI & H2RA	28

**Wheezing**		
Treated GERD		633
	PPI	143
	H2RA	454
	PPI & H2RA	36

Table S13. Severity of GERD. Adjusted hazard ratio and 95% CI. Pedianet, 2004-2015.

Clinical-**Asthma**	Nr· of Outcomes (N=106)	HRł	95% CI
Teated GERD	55	(Reference)	
Untreated GERD	51	1·25	(0·85 - 1·86)

**Treated-Asthma**	Nr· of Outcomes (N=242)	HRł	95% CI
Teated GERD	124	(Reference)	
Untreated GERD	118	1·01	(0·78 - 1·32)

**Wheezing**	Nr· of Outcomes (N=177)	HRł	95% CI
Teated GERD	89	(Reference)	
Untreated GERD	88	1·10	(0·81 - 1·5)

ł Adjusted for: Gender, Year of birth, Region, Number of antibiotic prescriptions in the first year of life, Number of outpatient visits

Figure S1. Definition of exposure and outcomes. Pedianet, 2004-2015.

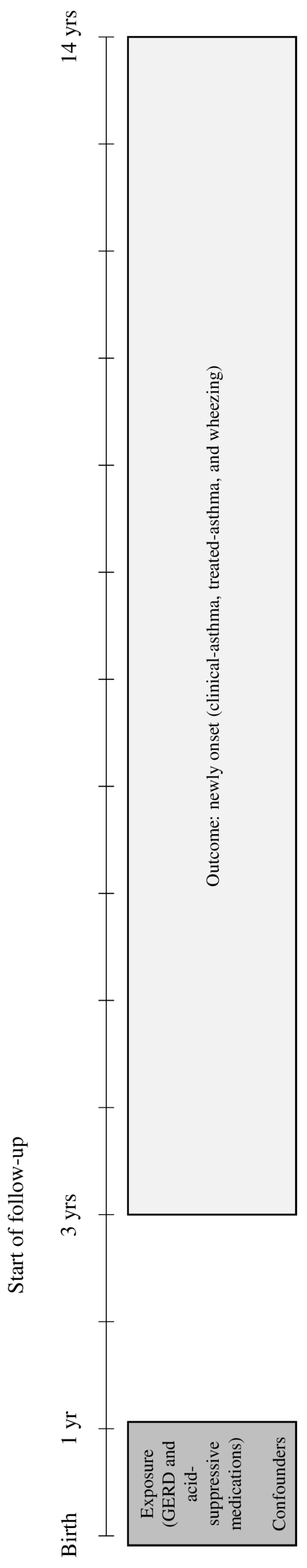

Figure S2. Nelson-Aalen cumulative hazards for the development of childhood asthma following exposure to GERD in the first year of life, compared to no GERD children.

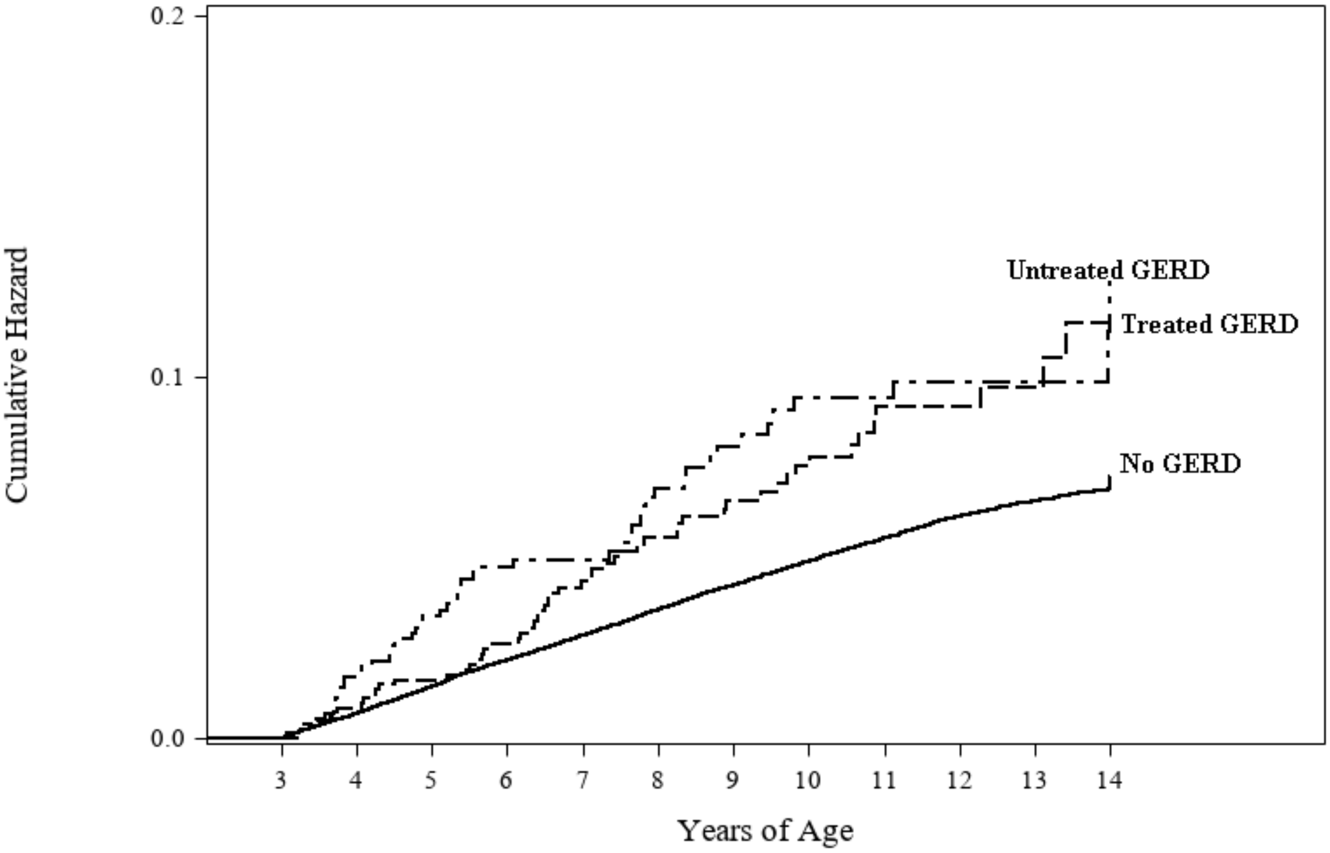

No GERD	83787	73943	65664	57401	48992	41559	34557	28117	22046	16265	11773
Treated GERD	856	784	732	660	578	505	436	348	249	175	115
Untreated GERD	768	667	589	499	431	357	297	240	199	164	115
